# Smart Sensing Chairs for Sitting Posture Detection, Classification, and Monitoring: A Comprehensive Review

**DOI:** 10.3390/s24092940

**Published:** 2024-05-05

**Authors:** David Faith Odesola, Janusz Kulon, Shiny Verghese, Adam Partlow, Colin Gibson

**Affiliations:** 1 Faculty of Computing, Engineering and Science, University of South Wales, Pontypridd CF37 1DL, UK; 30025293@students.southwales.ac.uk (D.F.O.); shiny.verghese@southwales.ac.uk (S.V.); 2 Rehabilitation Engineering Unit, Artificial Limb & Appliance Service, Cardiff and Vale University Health Board, Treforest Industrial Estate, Pontypridd CF37 5TF, UK; adam.partlow@wales.nhs.uk (A.P.); colin.gibson@wales.nhs.uk (C.G.)

**Keywords:** smart-sensing chair, musculoskeletal disorders, sitting posture classification

## Abstract

Incorrect sitting posture, characterized by asymmetrical or uneven positioning of the body, often leads to spinal misalignment and muscle tone imbalance. The prolonged maintenance of such postures can adversely impact well-being and contribute to the development of spinal deformities and musculoskeletal disorders. In response, smart sensing chairs equipped with cutting-edge sensor technologies have been introduced as a viable solution for the real-time detection, classification, and monitoring of sitting postures, aiming to mitigate the risk of musculoskeletal disorders and promote overall health. This comprehensive literature review evaluates the current body of research on smart sensing chairs, with a specific focus on the strategies used for posture detection and classification and the effectiveness of different sensor technologies. A meticulous search across MDPI, IEEE, Google Scholar, Scopus, and PubMed databases yielded 39 pertinent studies that utilized non-invasive methods for posture monitoring. The analysis revealed that Force Sensing Resistors (FSRs) are the predominant sensors utilized for posture detection, whereas Convolutional Neural Networks (CNNs) and Artificial Neural Networks (ANNs) are the leading machine learning models for posture classification. However, it was observed that CNNs and ANNs do not outperform traditional statistical models in terms of classification accuracy due to the constrained size and lack of diversity within training datasets. These datasets often fail to comprehensively represent the array of human body shapes and musculoskeletal configurations. Moreover, this review identifies a significant gap in the evaluation of user feedback mechanisms, essential for alerting users to their sitting posture and facilitating corrective adjustments.

## 1. Introduction

In 2020 alone, musculoskeletal disorders (MSDs) were ranked as the second leading cause of non-fatal disability, affecting over a billion people globally [1]. In the United Kingdom, more than 7.1 million adults suffer from MSDs, imposing an economic burden exceeding GBP 4.1 billion annually. Bevan et al. (2015) [2] highlighted that MSDs account for over 2% of the European Union’s gross domestic product (GDP), translating to an annual cost of approximately EUR 240 billion. These statistics underscore the increasing concern surrounding MSDs, necessitating effective interventions.

MSDs arise from a variety of factors, ranging from congenital defects [3] to neurological disorders [4]. Contrary to a common misconception, MSDs are not confined to the elderly; individuals of all ages are susceptible. The early development of MSDs can be attributed to various neurological conditions but also sedentary lifestyles and poor posture [5]. The office environment, characterized by prolonged periods of sitting, can exacerbate the risk of developing long-term musculoskeletal conditions, including back pain and spinal deformities [6,7,8]. Studies conducted among daily office workers concluded that there is a strong correlation between prolonged sitting and severe back pain affecting the lumbar area [9,10]. To combat this problem, a recommendation is that users take stroll breaks every few hours. The incorporation of exercise breaks as a daily routine potentially increases cognitive functions in the long term and improves muscle strength [11].

Poor sitting posture has long been recognized as a significant contributor to the development of pressure sores, adversely affecting the function, comfort, physiology, and mobility of individuals who use wheelchairs [12]. Healthcare professionals tasked with conducting postural assessments often rely on external observations to infer the internal configuration of musculoskeletal structures [13]. Typically performed in clinical settings, these assessments are subjective, with the detection of abnormalities dependent on visual inspection and palpation of the underlying skeletal structure [14]. Objective techniques for measuring musculoskeletal configuration such as MRI, CT scans, and X-rays are accurate but impractical for routine clinical use due to logistical, cost, and safety considerations, such as, notably, the risk of increased radiation exposure. Over the years, a diverse array of techniques for anthropometric measurements and postural assessments has been developed, broadly categorized into contact and non-contact methods. The contact methods include simple tactile devices such as anthropometric tapes, stadiometers, or scoliometers [15]. Non-contact techniques are radiography [16], Moiré fringe topography [17], structured light methods [17], laser scanning [18], pressure mapping systems [19], mechanical displacement sensors [20], and ultrasonic localization [21]. The primary drawbacks of tactile devices are their time-intensive nature, the absence of three-dimensional (3D) data, and potential discomfort for the patient. Non-contact methods, on the other hand, tend to offer enhanced accuracy and frequently provide 3D shape information. Yet, a significant limitation of these non-contact methods, particularly in the context of assessing sitting posture, is their dependence on direct access to the individual’s back. This necessitates the person to be in an upright, standing position for the measurement process, posing challenges for evaluations conducted in a seated posture.

Smart sensing chairs offer a solution to the limitations inherent in both contact and non-contact methods of assessing sitting posture. By integrating sensors directly into the seating surface and backrest, these chairs enable the continuous, real-time monitoring of posture without the need for direct physical contact or for the subject to be in a specific position, such as standing. Furthermore, the incorporation of smart sensing chairs into home or office environments enables active monitoring and feedback on a user’s health and activity levels. With the recent development in sensor technology and Artificial Intelligence, these systems hold promise for advancing personalized healthcare and enhancing quality of life, particularly for individuals afflicted with musculoskeletal disorders (MSDs).

The concept of a smart sensing chair was first explored by Tan et al. (2001) [22], pioneering the classification of sitting postures using integrated pressure sensors. Recent years have witnessed a surge in research focusing on smart sensing chairs, with approximately 500 studies published annually over the past five years. This trend underscores the growing interest in the field, highlighting the continuous increase in related publications.

The primary aim of this literature review is to evaluate published papers on smart sensing chair systems, aiming to understand the methods being employed in posture classification. By exploring existing studies, it is possible to analyze current trends such as the commonly used sensors and machine learning algorithms being adopted and potential research gaps. Ultimately, this review paper aims to provide valuable insight for researchers into the development of non-invasive smart sensing chair systems.

## 2. Research Methodology

This paper is aimed at conducting a comprehensive review of similar research studies conducted on smart sensing chair technology. Overall, there were 6 steps involved in the review process, which were the following: 1. Formulation of Research Questions, 2. Search Strategy, 3. Study Screening and Selection, 4. Data Extraction, 5. Discussion, 6. Conclusion and Recommendations.

### 2.1. Formulation of Research Questions

Table 1 presents the research questions for the comprehensive literature review on smart sensing chairs, each accompanied by its underlying rationale. These questions were crafted to guide the literature review of smart sensing chairs, targeting key aspects that are central to understanding the current state and future directions of this technology.

### 2.2. Search Strategy

A comprehensive search was conducted across several academic databases, including MDPI, IEEE, Google Scholar, Scopus, and PubMed, with the aim of finding relevant articles for this review. A predefined set of keywords and combinations thereof were used to refine the search, ensuring the retrieval of pertinent studies published in the last two decades. These search terms and phrases were specifically chosen to target topics related to smart sensing chairs and sitting posture classification, as detailed in Table 2. To enhance the search efficiency across databases, the terms were concatenated using the “OR” operator. The refined search string employed to query the relevant research databases was as follows: Smart Sensing Chair OR Sitting Posture Recognition OR Posture Classification OR Sitting Posture Classification Using Machine Learning OR Sitting Posture Monitoring OR Sitting Posture Detection.

### 2.3. Study Screening and Selection

**Figure 1:**
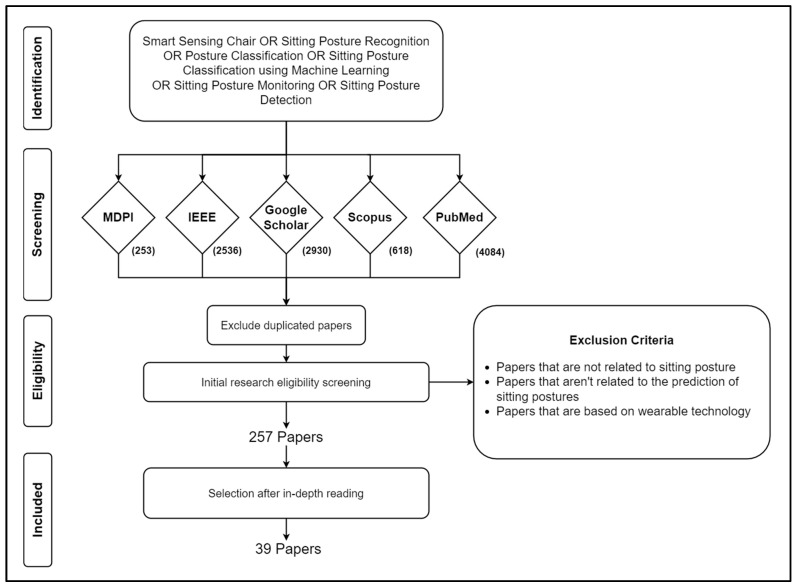
Literature review process.

The initial screening was based on the relevance of the titles and abstracts to the research questions. Studies published within the last 20 years were considered, applying the exclusion criteria to ensure that only relevant research studies were included in this comprehensive review. The initial database search yielded 253 papers from MDPI, 2536 from IEEE, 2930 from Google Scholar, 618 from Scopus, and 4084 from PubMed. To refine this selection and isolate relevant articles, specific conditions were applied to exclude studies that did not meet our criteria, including the following: 1. Papers unrelated to sitting posture, 2. Papers not concerning the prediction of sitting postures, and 3. Papers focused on wearable technology. The rigorous selection process, depicted in Figure 1, ultimately identified a total of 39 relevant papers.

### 2.4. Data Extraction

The data extraction phase was primarily focused on extracting relevant information from the research papers gathered. This was achieved by reading through each paper with the aim of gathering useful data, especially on the methods and techniques being employed in the development of smart sensing chair systems. Listed below is the following information that was captured while going through each research paper: Publication Year, Sensor Type Used, Number of Postures Classified and Recognized, Posture Classification Method, Classification Accuracy, Number of Test Subjects, and User Feedback Mechanism. Table 3 provides a list of all the research papers shortlisted, summarizing the key findings of each study.

## 3. Sitting Posture Selection

**Figure 2:**
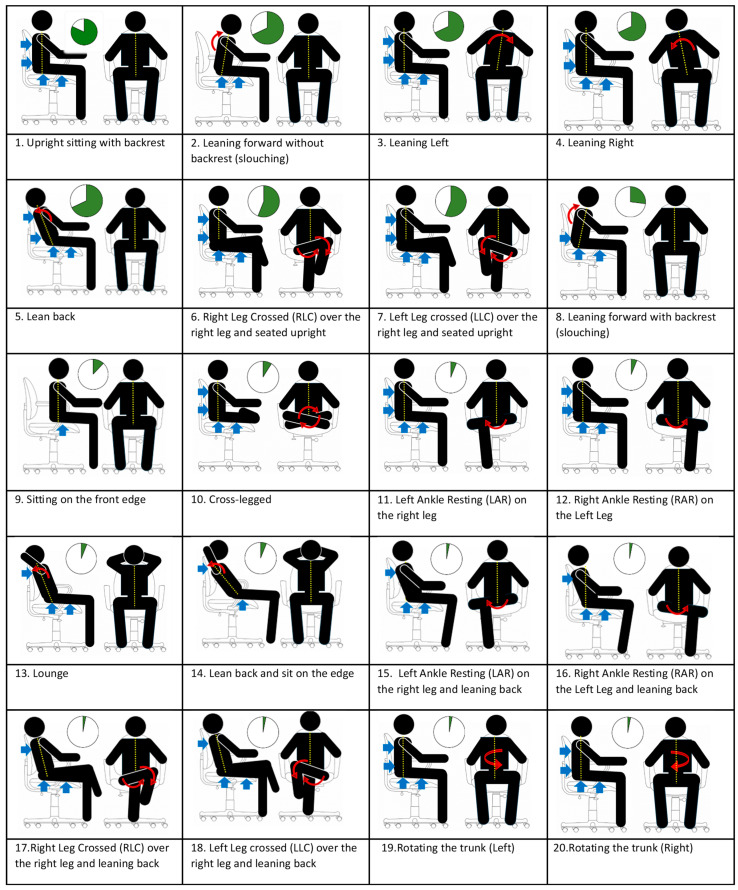
Twenty categories of different sitting postures along with a pie chart indicating their popularity among the research studies found.

The concept of an “ideal” sitting posture is inherently subjective, reflecting significant variation across diverse groups. Particularly for individuals with permanent mobility impairments or those who use wheelchairs, the parameters defining a comfortable sitting posture are distinctly unique. While the conventional wisdom among healthcare professionals advocates for an upright lordotic spinal position, the inherent variability in spinal anatomy across individuals challenges the notion of a one-size-fits-all “correct” posture [62]. Biomechanical research has shed light on the consequences of various sitting positions on spinal alignment and muscle engagement, emphasizing the musculoskeletal stress induced by inadequate postures [63]. These investigations reveal that extended periods of sitting, especially in a slumped position, intensify the symptoms of musculoskeletal disorders and are a contributing factor to lower back pain. Moreover, recommendations consistently suggest minimizing prolonged sitting durations, regardless of whether the posture is upright or slouched, to mitigate potential health risks. Korakakis et al. [63] underscored the absence of conclusive medical evidence associating any particular sitting posture with enhanced health benefits, further complicating the pursuit of an optimal sitting strategy. Figure 2 presents 20 sitting postures detected by smart sensing chair systems as reported in the literature, with the relative prevalence of each posture depicted through a pie chart. This pie chart quantifies the percentage of review papers that investigated each specific posture, ranking these postures from the most to the least frequently detected by such systems. Blue arrows highlight the primary pressure points at the interface between the seat and the occupant, while red arrows delineate the direction of adjustments necessary for adopting each specific posture. The most popular sitting postures detected by the smart sensing chairs included the following: 1. Upright sitting with backrest, 2. Leaning forward without backrest (slouching), 3. Leaning left, 4. Leaning right, and 5. Leaning back, reported by the majority of studies.

## 4. Technologies Used in Smart Sensing Chairs

### 4.1. Sensor Technology

**Figure 3:**
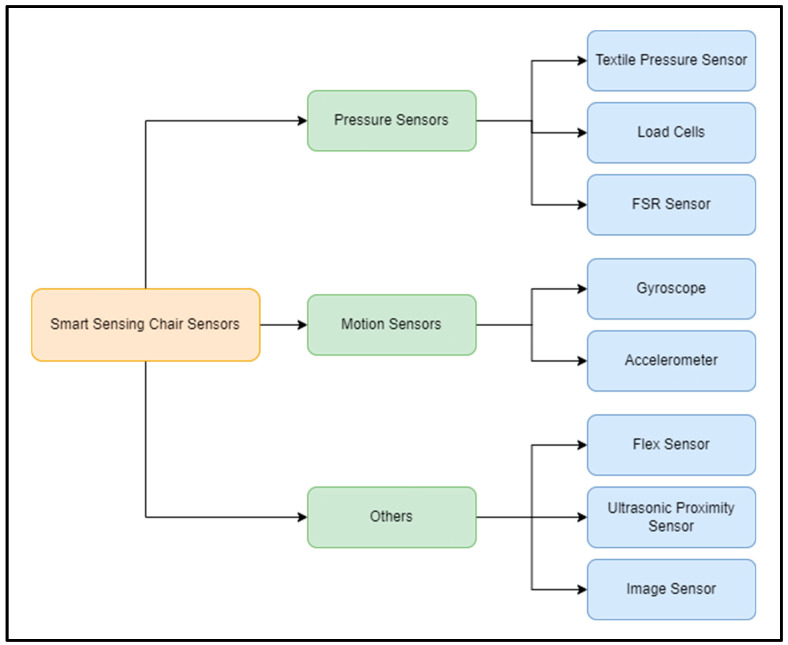
Taxonomy graph of sensors used in smart sensing chair systems.

Currently, there are several types of sensors being used in the development of smart sensing chairs, ranging from pressure sensors to image-based sensors. This section aims to review the variety of sensor technologies being integrated into smart sensing chair systems. Each offers unique benefits and challenges in the classification of sitting postures. Figure 3 below visualizes the category of sensors being used by researchers in the classification of sitting postures.

#### 4.1.1. Force Sensing/Sensitive Sensor (FSR)

**Figure 4:**
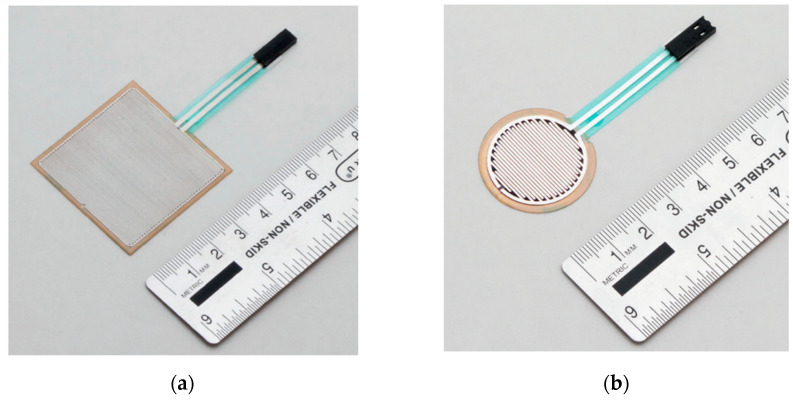
Examples of FSR sensors. (**a**) Square-shaped FSR sensor (FSR01CE) [67]. (**b**) Circle-shaped FSR sensor (FSR03CE) [67].

Force Sensing Resistors, also known as force sensors, are commonly used to measure the forces and physical pressure applied to a surface area. These sensors work by varying their output resistance in response to the pressure exerted on them. An FSR sensor, shown in Figure 4, is typically composed of a conductive polymer-based material that is integrated between two metal electrodes [64]. This conductive material changes in resistivity as more direct pressure is applied to the sensor’s z-axis. FSR sensors are also known to be very cost-effective and have been utilized in various fields ranging from robotics to medical applications [65]. However, the main limitation seen with these sensors is that they can be susceptible to drift errors, which can negatively affect the accuracy of their readings. Different methods such as periodical sensor calibration and other advanced force computing techniques are able to mitigate this issue [66]. Listed in Table 4 are some of the commercially available FSR sensors as well as some of their technical specifications.

#### 4.1.2. Textile Pressure Sensor

**Figure 5:**
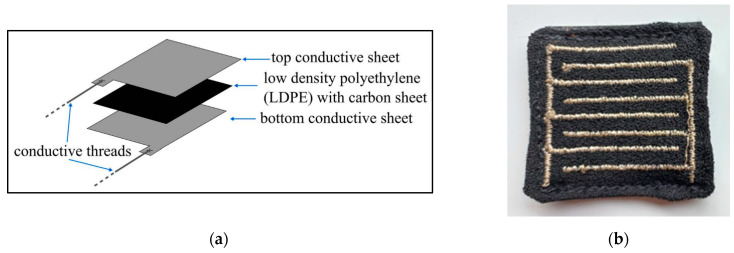
Textile pressure sensor. (**a**) Textile pressure sensor composition. Reproduced with permission [70]. (**b**) PreCaTex textile sensor. Reproduced with permission [26].

A textile-based pressure sensor is generally composed of a soft fabric-based material that consists of a conductive thread pattern placed over a dielectric material that serves as a substrate between the threads [70]. Figure 5a visualizes an example of how each layer within the textile pressure sensor is structured within. One of the main advantages seen with textile force sensors is the fact that they can be very durable and seamlessly integrate with garments, making them non-obstructive and comfortable for the end user. Thus, this sensor tends to be more popular among wearable technologies.

There were a few research studies found that employed textile pressure sensors to classify sitting postures. Kim et al. [32] developed a washable textile pressure sensor and incorporated it into their chair system to classify seven sitting postures using a decision-tree algorithm. Another study proposed an “eCushion” device that incorporated an “eTextile” pressure sensor array that could also detect seven different sitting postures with 85.90% accuracy [52]. Additionally, Martínez-Estrada et al. [26] developed 10 detachable textile pressure sensors (PreCaTex) (shown in Figure 5b) that were placed at strategic points around a chair.

#### 4.1.3. Load Cells

Load cells are another type of force sensor that is used among researchers in the monitoring of sitting postures. A load cell sensor works by converting applied mechanical forces into measurable digital signals that can be read by microcontrollers. There are different types of load cells being used such as strain gauge, piezoelectric, hydraulic, and capacitive load cells [71]. Some of the commercially available load cell sensors can be found in Table 5 below.

The use of load cells is not a popular option among research studies; only three research studies from this review implemented load cells in their smart sitting systems. Roh et al. in 2018 [31] developed a smart chair by integrating four load cell sensors within a chair sitting cushion to classify six sitting postures. An accuracy of 97.94% was achieved using an SVM (RBF kernel) ML model. Similarly, Pereira and Plácido da Silva in 2023 [23] distributed three load cells across a seat cushion in order to classify eight sitting postures; overall, they were able to achieve a classification accuracy of 98.50%. Tavares et al. [61] used four load cells along with four FSR pressure sensors to classify six different postures while achieving 100% accuracy.

#### 4.1.4. Flex Sensors

A flex sensor, also known as a bend sensor, works by measuring the degree of displacement resulting from the bending action being applied to the sensor. Currently, it is being used in various applications from robotics to medical devices. There are multiple types of flex sensors on the market; however, they are the conductive ink-based flex sensors that are widely popular among robotics projects. These flex sensors are typically composed of a flexible composite material that has a conductive ink material that changes in resistance as the sensor is bent [73]. Table 6 shows two commercially available flex sensors along with their technical specifications.

Overall, there were only two studies identified that utilized this method for sitting posture detection. The first study was by Hu et al. [34], who developed a smart sensing chair using six flex sensors and a two-layer Artificial Neural Network (ANN) for the detection of seven sitting postures while achieving an accuracy of 97.43%. The second study was by AbuTerkia et al. [51], who developed a similar smart seating system without the use of an ML model that could detect up to seven different sitting postures.

#### 4.1.5. Image-Based Sensors

Image-based sensors such as cameras and 3D image sensors are another type of technology being used by some studies that are often integrated with computer vision algorithms. These algorithms operate by identifying visual elements from images and videos. In the classification of sitting postures, there is typically a digital camera actively positioned directly at the subjects. Thus, with the use of image processing libraries such as OpenPose or OpenCV, researchers were able to analyze video frames to determine the sitting postures of test subjects in view.

This method was not a very popular option among the research studies found. Mallare et al. in 2017 [76] developed a system utilizing two digital cameras strategically positioned at (front and side) angles for the detection of bad sitting postures. Overall, they were only able to achieve an accuracy of 61.30% using the SVM algorithm. Additionally, Chen et al. in 2019 [45] further improved on this by using an Astra3D Sensor, a 3D depth-sensing camera. With the integration of the OpenPose library along with a CNN for posture classification, they were able to achieve an overall accuracy of 90%.

### 4.2. Pressure Sensor Placement Strategy

Across the research studies found, there were two main approaches employed in the placement of pressure sensors for smart sensing chair systems: dense sensor configuration and sparse sensor configuration, as described by Ma et al. [46]. Dense sensor configuration involves the use of a flexible sensor array mat containing multiple pressure sensor units interconnected together. Sparse sensor configuration works on the concept of having several individual pressure sensors placed at strategic points around a chair.

#### 4.2.1. Dense Sensor Configuration

**Figure 6:**
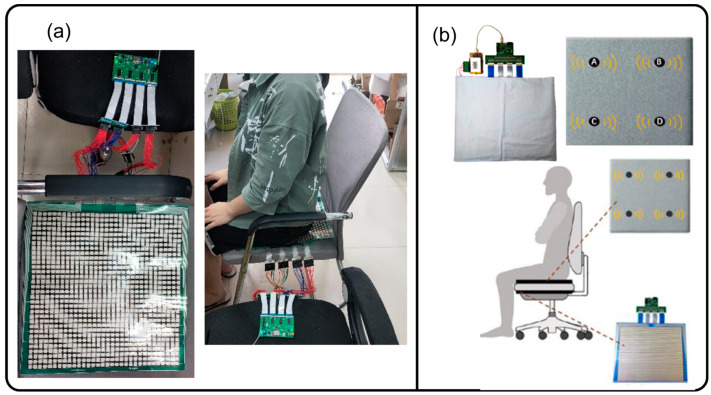
Illustration of some studies that implemented the use of dense sensor arrays. (**a**) Chair fitted with large pressure sensor array modules placed on top of the seating cushion. Reproduced with permission [43]. (**b**) Pressure array cushion with haptic feedback. Reproduced with permission [30], copyright 2021 *Sensors and Actuators*.

Xu et al. [52] used a textile pressure sensor array along with a dynamic time wrapping-based algorithm to classify seven sitting postures with 85.90% accuracy. Huang et al. [25] used a 52 × 44 Piezo-Resistive Sensor Array that was placed on the surface of the seating area. Using the ANN classifier, they were able to achieve a classification accuracy of 92.2%. Kim et al. in 2018 [32] developed a washable fabric-based sensor array. Even after one thousand independent washes, the capacitance reading from the textile sensor array did not deteriorate. Kim et al. [41] achieved 95.30% accuracy using an 8 × 8 pressure array and a CNN classifier to classify five sitting postures among children. Similarly, Cai et al. [43] utilized a flexible pressure sensor array (400 mm × 400 mm) placed on a bottom seat cushion to recognize six different sitting postures, as shown in Figure 6a. Ran et al. [30] installed an 11 × 13 Pressure Sensor Array (IMM00014, I-MOTION) that communicated with a Raspberry PI computer, achieving 96.22% classification accuracy using a five-layer ANN classifier, seen in Figure 6b. Ahmad et al. [24] embedded a 16-screen pressure sensor array, also using a Raspberry Pi computer for sitting classification, which obtained a high accuracy of 99.03% using the LightGBM machine learning algorithm. Wang et al. [48] developed two sets of interconnected sensor sheets that covered both the backrest and the seating cushion of a smart sensing chair. Using the SNN classifier, their proposed system could distinguish 15 different sitting postures with an accuracy of 88.52%, among the highest number of postures being classified. Fan et al. [44] also implemented a similar system that analyzed hip pressure, which subsequently achieved an accuracy of 99.82% using a CNN. Table 7 below provides a list of studies that used sensor arrays for the classification of sitting postures.

#### 4.2.2. Sparse Sensor Configuration

**Figure 7:**
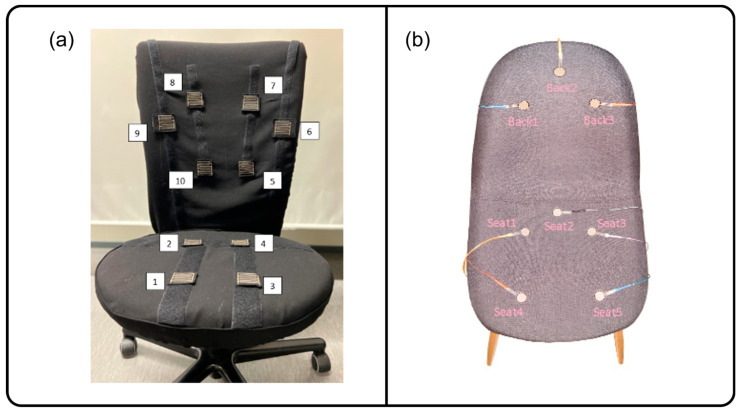
Research studies using multiple pressure sensors placed around a chair. (**a**) Chair fitted with 10 textile pressure sensors. Reproduced with permission [26]. (**b**) Eight FSR sensors placed around a chair, five sensors placed on a sitting cushion, and three sensors added to a backrest. Reproduced with permission [28].

This sensor configuration appears to be a more popular option as more studies implemented this setup compared to dense sensor configuration. Mutlu et al. in 2007 [37] integrated 19 different FSRs into a seating cushion and used the Simple Logistic Regression ML algorithm to achieve 78% accuracy in classifying 10 different postures. Martínez-Estrada et al. [26] placed six textile sensors on a backrest and an additional four sensors on a seating cushion in order to classify eight sitting postures, as shown in Figure 7a. Tsai et al. [40] used 13 pressure sensors to classify 10 sitting postures and was able to achieve an accuracy of 99.10% using the SVM algorithm. Aminosharieh Najafi et al. [28] applied eight sensors (four on the seating cushion and four on the backrest) and used the EMN algorithm to classify eight sitting postures, and achieved an accuracy of 91.68%, as seen in Figure 7b. In addition to this, a Desktop Graphical User Interface (GUI) application was also developed, which displayed a sensor reading in real-time. Luna-Perejón et al. [42] added six sensors that were placed on a seating cushion, resulting in an 81.50% classification accuracy using the SOM (ISOM-SPR) ML algorithm. Table 8 below provides a full list of studies using this sensor configuration.

### 4.3. Integration with the Internet of Things (IoT)

Over recent years, IoT has gained in popularity and become a game changer within certain industries. It was projected that by the year 2030, there would be over 50 billion devices interconnected through the IoT [77]. Within the context of smart sensing chair systems, Ma et al. [46] highlighted the effectiveness of integrating IoT-based systems into healthcare sensor systems due to their major advantage of being able to seamlessly monitor users’ health data in real-time. The use of IoT systems for remote health monitoring is believed to not only reduce medical costs but also aid in the early detection of chronic illnesses. Subsequently, this could potentially accelerate treatment and improve the overall life expectancy of an individual.

There were some studies found that utilized IoT-based technology. Matuska et al. [27] used an Arduino-based microcontroller that communicated using the Message Queuing Telemetry Transport (MQTT) telemetry protocol in the detection of nine different sitting postures. The sensor data was sent in real-time to a mobile application that alerted a user if an incorrect posture was being detected by displaying “green”, “orange”, and “red” for standard sitting, bad sitting, and heavy load on backbone, respectively. Other studies such as [53,55] similarly used IoT for bad posture detection and to provide valuable, real-time feedback to the end-user.

### 4.4. User Feedback System

The integration of a feedback system into a smart sensing chair is an integral component of enhancing the user experience. From the end user’s perspective, individuals should be able to receive real-time alerts whenever an improper sitting posture is detected. It was seen that most studies focused on the classification aspects and left out the implementation of a feedback platform. Only 36% (14) of all the studies incorporated a feedback platform to encourage users to maintain a correct posture. The implementation of mobile applications was seen as the most used platform for alerting a user whenever an improper sitting posture was detected [27,36,43,54]. Another common method was the use of a desktop application, which was observed in some studies [40,45,48,53]. Ran et al. [30], on the other hand, proposed the use of a haptic motor system that was integrated into a seating cushion and vibrated its motors whenever an incorrect sitting posture was detected. To make the system as unintrusive as possible, another study by Ren et al. [47] looked at using an RGB bulb capable of changing color whenever an incorrect posture was detected.

## 5. Techniques for Posture Detection in Smart Sensing Chairs

Posture detection is a critical function of smart sensing chair systems. This section explores the diverse techniques and machine learning algorithms employed to classify various sitting postures, which range from traditional rule-based approaches through statistical methods to advanced machine learning models.

### 5.1. Rule-Based Systems

Rule-based systems operate by employing a series of if–else conditions or predefined rules to make decisions based on sensor inputs [78]. In the context of posture classification, these systems are configured to establish specific data thresholds during the testing phase, which are used to align sensor readings with corresponding sitting postures. Our review documented several studies that effectively utilized rule-based systems for posture recognition [26,27,32,61].

The primary advantage of rule-based systems lies in their transparent and explainable decision-making process. These systems are highly efficient, requiring minimal computational resources, which renders them particularly suitable for real-time applications [79]. Additionally, their straightforward logic and operational mechanisms allow for ease of understanding and implementation. However, as the complexity of posture detection increases, rule-based systems can become unwieldy [27]. The number of rules needed to cover all potential sitting postures and transitions can grow significantly [61], making the system difficult to manage and prone to errors. Scalability issues also arise as managing and updating a large set of rules for various postures can become cumbersome. This challenge can be magnified in healthcare settings, where the variety of user body shapes and complex muscular skeletal configuration broadens the scope of posture detection.

### 5.2. Statistical Models

Statistical models analyze data by identifying correlations between variables and making predictions based on statistical hypotheses derived from a dataset [80]. Various statistical models have been used to classify sitting postures, including K-Nearest Neighbors (KNNs) [23,35,57,60], Decision Tree [38,46,50], Support Vector Machines (SVMs) [29,31,40], Random Forests (RFs) [33,39,55], Light Gradient-Boosting Machine (LightGBM) [24,58], Simple Logistic Regression (SLR) [37], and Self-Organizing Maps (SOMs) [43]. In the context of smart sensing chairs, statistical models offer a balance between complexity and performance, making them integral tools for posture classification. They are also less resource-intensive compared to more complex models [33], making them well-suited for real-time processing in embedded systems. However, the choice of model should be aligned with the specific characteristics of the posture data and the operational demands of the smart chair system to optimize both user comfort and system efficiency.

### 5.3. Deep Learning Models

Deep learning models [81], characterized by their multi-layered neural network structure that includes an input layer, several hidden layers, and an output layer, have been extensively employed in the classification of sitting postures in smart sensing chairs. Research has primarily focused on utilizing Convolutional Neural Networks (CNNs) [41,44,45,49,54], Artificial Neural Networks (ANNs) [25,30,34,36,42,47], Spiking Neural Networks (SNNs) [48], and Deep Neural Networks (DNNs) [59] for this purpose. Both CNNs and ANNs have emerged as particularly popular choices due to their robustness in handling complex pattern recognition tasks. Deep learning models are advantageous for posture classification in smart sensing chairs due to their ability to process large datasets and perform automatic feature extraction, essential for analyzing the spatial arrangement of pressure points on a chair. However, these models require extensive, well-labeled training data to function effectively without overfitting [82]. A lack of diverse data can lead to biases, limiting their real-world applicability, particularly in varied clinical settings. Additionally, the complex nature of these models often results in a “black box” phenomenon, making it difficult to interpret decision-making processes, which is crucial in medical applications where transparency is necessary.

### 5.4. Evaluation of Machine Learning Model performance

The accurate evaluation of machine learning models is essential for validating their performance and accuracy. Commonly used methods include confusion matrices and performance comparisons between different models. A confusion matrix helps measure algorithm performance, indicating True Positives (TPs), True Negatives (TNs), False Positives (FPs), and False Negatives (FNs) for binary classification, while extending to an NxN matrix for multi-class models, where N represents the number of classes [83].

## 6. Discussion

### 6.1. Technology

**Figure 8:**
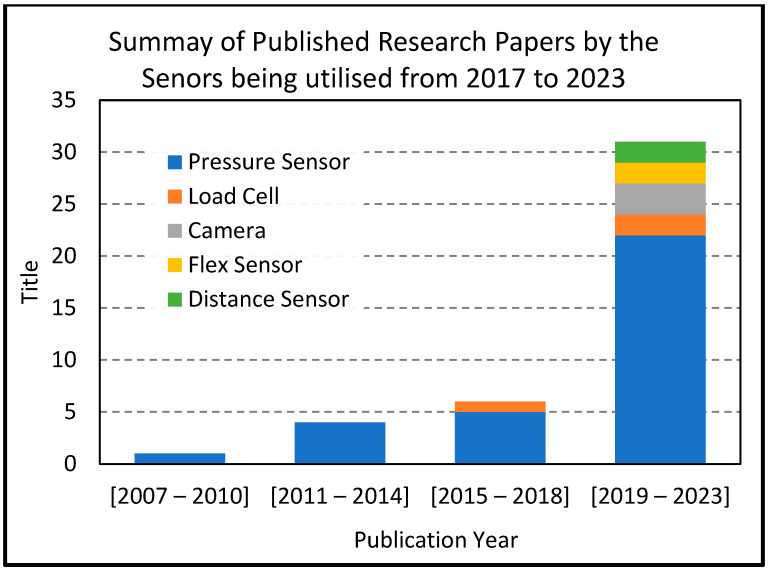
Number of research papers published on smart sensing chair technology along with the sensor being used from 2007 to 2023.

The vast majority of the research studies revealed that the most popular approach to developing a smart sensing chair was to employ the use of pressure sensors. Figure 8 clearly shows that over the years, pressure sensors have always been the preferred option for the classification of sitting postures among researchers, among which FSR sensors were the preferred option compared to textile pressure sensors.

In terms of the sensor placement configuration, placing various individual pressure sensors around a chair tends to be the preferred method, rather than utilizing dense pressure arrays. There was no correlation seen that suggested that one sensor placement strategy produced higher classification accuracy than another. However, there are other variables that should be considered such as maintenance and costs. Dense sensor arrays are known to be more costly and harder to manage compared to their counterparts [46]. This is because if one or more of the individual sensing units within an array were faulty, the entire sensor array would need to be replaced, further increasing maintenance costs.

#### Multiple Sensor Types

While most studies utilized a singular type of sensor for posture detection, there were only a few studies that involved multiple sensor types in their proposed smart chair system. Jeong and Park [35] utilized six pressure sensors (placed on the seating cushion) along with six Infrared Reflective Distance Sensors (placed on the backrest). By using the K-Nearest Network (KNN), they were able to classify 11 different sitting postures while achieving an accuracy of 92%, compared to 59% while using only pressure sensors. This study highlighted one of the main limitations seen with other smart sensing systems, where pressure sensors alone are incapable of measuring the spinal trunk angle, which is yet another important factor in maintaining a proper sitting posture. Similarly, Cho et al. [54] used 16 pressure sensors placed on the sitting cushion along with two ultrasonic sensors placed at the neck support region. With this configuration, they were able to achieve 96% accuracy using the Lower-Balance Check Network (LBCNet) to classify 15 sitting postures.

Furthermore, integrating multiple sensor types to enhance sitting posture classification improves classification accuracy by expanding sensor coverage, thereby increasing the system’s robustness [35]. This integration also offers additional benefits beyond basic posture detection, such as continuous health monitoring and rehabilitative support. For example, a recent study by Pereira et al. [23] demonstrated the potential for invisible electrocardiography (ECG) monitoring using conductive Nappa placed strategically at the armrests.

However, this approach also presents several challenges. Data fusion complexity is a significant hurdle, as combining multiple types of sensors often necessitates advanced data fusion techniques, particularly in IoT-based devices [84]. The cost of using multiple sensors also introduces financial considerations, including potential increases in system maintenance expenses over time. Additionally, the collection and storage of sensitive user data raises important concerns about data privacy and security. It is crucial to ensure that robust security measures are in place and that data handling complies with privacy regulations [85].

### 6.2. Classification Algorithm

**Figure 9:**
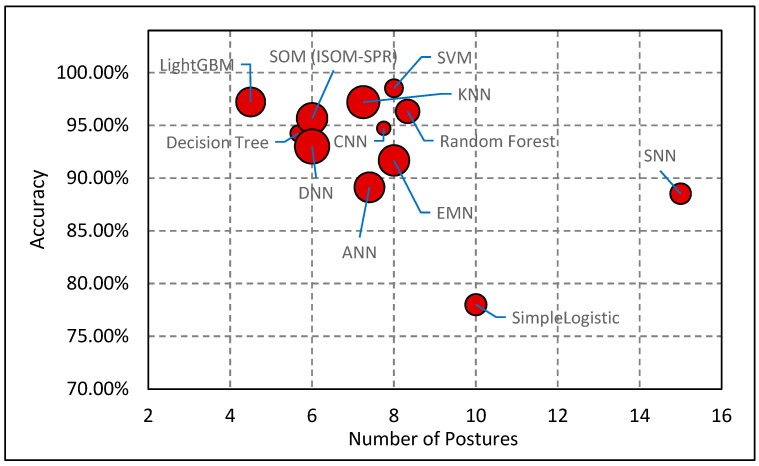
Comparison of machine learning models: number of postures vs. accuracy vs. test subjects, as indicated by the size of the circle.

Figure 9 illustrates the relationship between the number of sitting postures classified and the overall classification accuracy of various machine learning models, as reported in the literature. Data analysis indicated a moderate negative correlation between a model’s accuracy and the number of postures it classified. This trend indicates that as the complexity of posture classification increases—with more postures being identified—the precision of classification tends to decrease. Consequently, this pattern has led researchers to typically restrict the scope of posture detection to between five and seven specific positions, including leaning left, leaning right, leaning backward, upright sitting, and leaning forward, to optimize accuracy. A study by Feng et al. [33] that utilized RFID tags in conjunction with a camera sensor to classify three distinct sitting postures—sitting straight, leaning forward, and leaning backward—representing the lower end of the posture classification spectrum. In contrast, investigations by Wang et al. [48], Cho et al. [54], and Bourahmoune et al. [55] expanded posture classification to encompass up to 15 different postures, achieving notable accuracies of 88.52%, 96%, and 98.82%, respectively. This range highlights the diverse capabilities and limitations of machine learning applications in posture detection within smart sensing chair systems.

Figure 9 also indicates that deep learning models, including Convolutional Neural Networks (CNNs) and Artificial Neural Networks (ANNs), do not significantly outperform traditional statistical models in terms of classification accuracy for sitting posture detection. This observation may be attributed to the size of the datasets employed for model training. Deep learning models are recognized for their superior performance with extensive datasets, in contrast to statistical models that require less data. An additional reason for this disparity is the limited number of test subjects contributing data to train the deep learning models, suggesting that an expansion of dataset size could enhance their classification accuracy.

### 6.3. Research Gaps

#### 6.3.1. Lack of User Feedback Evaluation

In examining the current state of this research field, many of the studies predominantly focused on the development of algorithms that would achieve high classification accuracy. Although the pursuit of enhanced algorithmic performance in posture detection is important, there exists a noticeable void in the integration and subsequent evaluation of user feedback methods. Most studies tended to prioritize other aspects such as sensor placement and classification accuracy and leave out critical evaluations of user feedback systems for posture correction. As previously discussed, only 14 studies implemented a user feedback system for posture correction, 6 of which used a mobile application. This limited adoption underscores a significant research gap in the assessment of such feedback systems.

With the lack of comprehensive evaluations being conducted, a few questions can be raised regarding effectiveness, feasibility, and overall usability from the end user’s perspective when interacting with these systems. Are these systems truly effective in motivating and guiding users towards adopting healthier sitting postures? Performing a critical evaluation of these systems would be beneficial in various aspects. Firstly, it would provide vital information regarding the user experience while interacting with these systems, making it quite easy to find potential gaps that could be further improved upon. Moreover, a detailed examination would elucidate whether user expectations align with a system’s outcomes, thereby facilitating targeted improvements to ensure agreement between user needs and system functionality. Employing methodologies such as user interviews, surveys, and usability testing stands to offer invaluable feedback, paving the way for the refinement of feedback mechanisms within smart sensing chair systems.

#### 6.3.2. Lack of Diversity of Training Datasets

The quality of the training dataset is very important during the training of a machine learning model. In the process of model training, test subjects are commonly enlisted to simulate various sitting postures over designated periods. On average, the research studies utilized a low number of test subjects, typically around 21 individuals. A sample size this small might not be adequate to fully represent the wide postural variances that exist within the wider population. Additionally, there also seemed to be a bias towards the test subjects involved in data collection, most of whom were healthy individuals mocking poor sitting postures.

While this no doubt simplifies the data collection phase for most studies, it fails to account for the different challenges involved in the recognition of poor sitting postures among individuals suffering from musculoskeletal conditions. Consequently, the effectiveness of the machine learning model might be compromised when applied in real scenario settings involving a much wider demographic.

Addressing this issue requires a lot of effort that involves broadening datasets by the inclusion of a wider demographic with different age groups, body shapes, and health conditions. Enriching the datasets in this manner would enhance the models’ ability to accurately classify sitting postures among a heterogeneous population, thereby increasing their robustness and applicability in diverse real-world scenarios.

### 6.4. Feasibility of Implementing Smart Sensing Chair Systems in Real-World Settings

The implementation of smart sensing chairs in real-life scenarios such as offices or healthcare facilities has both opportunities and challenges. The current advancement of sensor technology has made it increasingly possible to actively monitor various sitting postures while also providing valuable user feedback in real-time. As previously discussed, smart sensing chair systems have the capacity to promote better sitting and posture habits by reducing the risk of musculoskeletal disorders among individuals who are regularly seated for an extended period, further improving quality of life by actively promoting the habit of a wellness attitude while in the workplace of the healthcare environment. However, the implementation of smart sensing chairs also involves several challenges. For these systems to be successful, they must be embraced by users, including employees in office settings and patients in healthcare facilities. Promoting effective user adoption can be achieved through training sessions that emphasize the health benefits of smart chairs and by designing interfaces that are intuitive and encourage frequent use. One primary concern is the reliability and accuracy of sensor data, including the potential for false positives in posture detection. Sensor drift represents a recurring risk, which can lead to data inaccuracies over time [86]. The regular calibration of sensors is crucial to maintain their accuracy and effectiveness in interpreting users’ postures.

Integration with existing technological infrastructures also poses significant challenges. Smart sensing chairs need to be compatible with workplace networks and healthcare IT systems without requiring extensive modifications. The rise of IoT-based technologies offers feasible solutions for integration within these environments [87,88,89]. It is vital that these systems comply with the communication and security protocols of the respective organizations.

Maintenance overheads and compatibility with existing furniture and wheelchair systems are additional concerns. Furthermore, the implementation of these systems must address data privacy issues related to the collection of sensitive user information. Regarding cost-effectiveness, the affordability of a system is heavily influenced by the cost of hardware components, particularly sensors and computing units. Each of these factors must be carefully considered to ensure that smart sensing chairs are a viable and beneficial addition to both office and healthcare settings.

## 7. Conclusions and Recommendations for Future Research

This paper provided a comprehensive literature review of smart sensing chair systems within the research landscape. It identified a diverse array of sensors utilized across studies, including Force Sensing Resistors (FSRs), textile pressure sensors, load cells, and image sensors, with FSR sensors emerging as the predominant choice among researchers. The strategies for sensor placement predominantly fall into two categories: utilizing a pressure sensor array or distributing individual sensors throughout a chair. Presently, no conclusive evidence suggests a definitive advantage of one strategy over another in terms of enhancing classification accuracy. However, from maintenance and cost perspectives, the dispersed sensor approach is deemed more favorable but may not be feasible for people with MSDs due to their unique and complex body shapes. In the area of sitting posture classification, various machine learning models have been employed, with many achieving a high classification accuracy rate of 90%. Despite these successes, a notable gap in the research is the quality of the datasets used for training these models. Predominantly, test subjects are healthy individuals from a narrow demographic simulating incorrect sitting postures, which raises concerns about these models’ applicability to broader populations, particularly those with musculoskeletal disorders.

Looking ahead, it is important for future research to prioritize the development and rigorous evaluation of user feedback systems aimed at posture correction. Such investigations would significantly contribute to assessing the effectiveness of these systems in real-world settings. Validation tools such as the System Usability Scale, NASA Task-Load Index (TLX), and Single Ease Questions could be implemented to assess user usability across an entire system [90,91].

Moreover, there is a compelling case for exploring the integration of various sensor types to enhance the functionality of smart sensing chair systems. While current studies often focus on a single sensor type for posture detection, the integration of multiple sensor types, as demonstrated by Jeong and Park [35], who combined infrared reflective distance sensors with pressure sensors, could offer a more versatile approach to posture classification. Incorporating Inertial Measurement Unit (IMU) sensors could further enable the monitoring of user activity, enriching the data available for posture analysis and correction [46].

## Figures and Tables

**Table 1:** Research questions.

ID	Research Question and Rationale
RQ1	In the context of posture detection, what are the most used sensors in smart sensing chair studies, and how do they compare in terms of accuracy and reliability? Rationale: This question aimed to uncover common trends in sensor technology that can inform the development of more effective and sensitive smart chairs for posture detection.
RQ2	What methods are being used to classify different sitting postures? Rationale: This question addressed the computational approaches employed to process sensor data, which is essential for the effective classification of sitting postures. Understanding the methods used can highlight the most successful strategies and potential areas for innovation in posture classification algorithms.
RQ3	What technological, methodological, and application-based limitations and research gaps are identified in the current literature on smart sensing chairs? Rationale: This question sought to pinpoint the shortcomings of current studies on smart sensing chairs, laying the groundwork for future research to address these areas.
RQ4	What user feedback mechanisms are implemented in smart sensing chairs, and how do they impact user satisfaction and posture correction outcomes? Rationale: The incorporation of user feedback mechanisms is critical for the practical application of smart sensing chairs, influencing user compliance and the effectiveness of posture correction strategies. This question focused on the interaction between users and the technology, a key factor in the adoption and success of these systems.

**Table 2:** List of search keywords.

ID	Keywords
SK1	Smart Sensing Chair
SK2	Sitting Posture Recognition
SK3	Posture Classification
SK4	Sitting Posture Classification Using Machine Learning
SK5	Sitting Posture Monitoring
SK6	Sitting Posture Detection

**Table 3:** Summary of the shortlisted papers.

Study	Publication Year	Sensor Type	# of Postures	Classification Method	Accuracy	# of Test Subjects	User Feedback Mechanism
[23]	2023	Load Cell	8	KNN	98.50%	10	-
[24]	2021	Pressure Sensor	4	LightGBM	99.03%	32	-
[25]	2017	Pressure Sensor	8	ANN	92.20%	-	-
[26]	2023	Pressure Sensor	9	-	-	5	-
[27]	2020	Pressure Sensor	9	-	-	12	Mobile App
[28]	2022	Pressure Sensor	8	EMN	91.68%	40	-
[29]	2021	Pressure Sensor	12	SVM	89.60%	3	-
[30]	2021	Pressure Sensor	7	ANN	97.07%	100	Haptic Feedback
[31]	2018	Load Cell	6	SVM	97.94%	9	-
[32]	2018	Pressure Sensor	7	-	-	-	-
[33]	2019	Camera and RFID Tags	3	RF	99.27%	14	-
[34]	2020	Flex Sensor	7	ANN	97.43%	11	-
[35]	2021	Pressure Sensor and Ultrasonic Sensor	11	KNN	92%	36	-
[36]	2013	Pressure Sensor	8	ANN	70%	30	Mobile App
[37]	2007	Pressure Sensor	10	SimpleLogistic	78%	20	-
[38]	2017	Pressure Sensor	5	DT	99.47%	12	-
[39]	2016	Pressure Sensor	7	RF	90.90%	41	-
[40]	2023	Pressure Sensor	10	SVM	99.10%	20	Desktop App
[41]	2018	Pressure Sensor	5	CNN	95.30%	10	-
[42]	2021	Pressure Sensor	7	ANN	81%	12	Desktop App
[43]	2021	Pressure Sensor	6	SOM (ISOM-SPR)	95.67%	40	Mobile App
[44]	2022	Pressure Sensor	5	CNN	99.82	8	-
[45]	2019	Camera	-	CNN	90%	-	Desktop App
[46]	2020	Pressure Sensor	5	DT	89%	-	-
[47]	2019	Pressure Sensor	-	ANN	-	-	RGB LED
[48]	2021	Pressure Sensor	15	SNN	88.52%	19	Desktop App
[49]	2023	Camera	6	RCNN and CNN	92.50%	-	-
[50]	2014	Pressure Sensor	7	DT	-	-	-
[51]	2022	Flex Sensor	7	-	-	-	-
[52]	2013	Pressure Sensor	7	Dynamic Time Warping	85.90%	14	-
[53]	2023	Pressure Sensor	6	-	-	2	Desktop App
[54]	2019	Pressure Sensor and Ultrasonic Sensor	15	CNN and LBCNet	96%	8	Mobile App
[55]	2022	Pressure Sensor	15	RF	98.82%	18	Mobile App and Haptic Feedback
[56]	2023	Pressure Sensor	6	-	95%	37	Desktop App
[57]	2019	Pressure Sensor	5	KNN	98.33%	12	-
[58]	2022	Pressure Sensor	5	LightGBM	95.41%	40	-
[59]	2022	Pressure Sensor	6	DNN	93%	50	-
[60]	2023	Pressure Sensor	5	KNN	99.99%	118	-
[61]	2023	Pressure Sensor and Load Cell	6	-	100%	6	Mobile App and Desktop App

In this table, the “-“ symbol denotes “data not included” for the respective category.

**Table 4:** Technical specifications of commercially available FSR sensors.

Model	Manufacturer	Dimensions (Length × Width × Thickness) (mm)	Force Sensitivity Range (Newtons)
FSR 402 [68]	Interlink Electronics	14.68 × 14.68 × 0.46	0.1–100 N
FSR 406 [69]	Interlink Electronics	39.60 × 39.60 × 0.46	0.1–100 N
FSR01CE [67]	Ohmite	39.70 × 39.70 × 0.375	Up to 49 N

**Table 5:** Technical specifications of commercially available load cell sensors.

Model	Manufacturer	Dimensions (Length × Width) (mm)	Capacity (kg)
SEN-10245 [72]	SparkFun Electronics	34 × 34	40–50
P0236-142 [31]	Hanjin Data Corps	34 × 34	-

**Table 6:** Technical specifications of commercially available flex sensors.

Model	Manufacturer	Dimensions (Length × Width) (mm)	Flat Resistance
FS-L-055-253-ST [74]	Spectra Symbol	112.24 × 6.35	10 K Ohms
Flex Sensor 2.2 [75]	Spectra Symbol	73.66 × 6.35	25 K Ohms

**Table 7:** Studies using dense sensor array.

Sensor	Accuracy	# of Postures
Textile Pressure Sensor Array [52]	85%	14
52 × 44 Piezo-Resistive Sensor Array [25]	92%	8
32 × 32 Pressure Sensor Array [29]	89.60%	4
Textile Pressure Sensors (Woven Fabric) [32]	-	7
8 × 8 Pressure Mat Sensor [41]	95%	5
400 mm × 400 mm Flexible Array Pressure Sensor [43]	95%	6
11 × 13 Pressure Array (IMM00014, I-MOTION) [30]	97%	7
Screen-Printed Pressure Sensor Units (16-Array) [24]	99%	4
Two Pressure Sensor Arrays (FSR) [48]	88%	15
44 × 52 Pressure Sensor Array [44]	99%	5
32 × 32 Pressure Sensor Array [59]	93%	6

**Table 8:** Studies using sparse sensor array configuration.

Sensor	Accuracy	# of Postures
10 Textile Capacitive Sensors (PreCaTex) [26]	-	8
19 4 × 4 Pressure Sensors (Force Sensing Resistors) [37]	78%	10
6 Flexible Force Sensors (FSR402) [27]	-	9
8 Force Sensing Resistors [28]	91%	8
6 Pressure Sensors and 6 Infrared Reflective Distance Sensors [35]	92%	11
8 Low-Resolution Matrices of Pressure Sensors [36]	70%	8
12 Pressure Sensors (Force Sensitive Resistor) [38]	99%	5
16 Force Sensors and Accelerometer [39]	90%	7
13 Pressure Sensors (FSR-406) [40]	99%	10
6 Force Sensitive Resistors (FSRs) [42]	81%	7
6 FSR Sensors [46]	89%	5
6 Square-Type Force Sensing Resistors [47]	-	-
8 Force Sensing Resistors FSR 406 [50]	-	7
5 Flex Sensors [51]	-	7
4 FSR Pressure Sensors [53]	-	6
16 Pressure Sensors and 2 Ultrasonic Sensors [54]	96%	15
9 E-Textile Pressure Sensors [55]	98%	15
9 FSR Sensors [60]	99%	5
4 FSR Sensors and 4 Load Cells [61]	100%	6
9 FSR Sensors [58]	95%	5
13 Piezoresistive Sensors [57]	98%	5
16 FSR Sensors [56]	95%	6

## Data Availability

No new data were created or analyzed in this study. Data sharing is not applicable to this article.
